# A Noninvasive Test for MicroRNA Expression in Oral Squamous Cell Carcinoma

**DOI:** 10.3390/ijms19061789

**Published:** 2018-06-16

**Authors:** Davide B. Gissi, Luca Morandi, Andrea Gabusi, Achille Tarsitano, Claudio Marchetti, Francesca Cura, Annalisa Palmieri, Lucio Montebugnoli, Sofia Asioli, Maria P. Foschini, Luca Scapoli

**Affiliations:** 1 Department of Biomedical and Neuromotor Sciences, Section of Oral Sciences, University of Bologna, 40159 Bologna, Italy; davide.gissi@unibo.it (D.B.G.); andrea.gabusi3@unibo.it (A.G.); lucio.montebugnoli@unibo.it (L.M.); 2 Department of Biomedical and Neuromotor Sciences, Section of Anatomic Pathology at Bellaria Hospital, University of Bologna, 40139 Bologna, Italy; sofia.asioli3@unibo.it (S.A.); mariapia.foschini@unibo.it (M.P.F.); 3 Department of Biomedical and Neuromotor Sciences, Section of Maxillo-Facial Surgery at Policlinico S. Orsola-Malpighi, University of Bologna, 40138 Bologna, Italy; achille.tarsitano2@unibo.it (A.T.); claudio.marchetti@unibo.it (C.M.); 4 Department of Experimental, Diagnostic and Specialty Medicine, University of Bologna, 40126 Bologna, Italy; francesca.cura@unife.it (F.C.); annalisa.palmieri@unibo.it (A.P.); luca.scapoli2@unibo.it (L.S.)

**Keywords:** oral squamous cell carcinoma, field cancerization, diagnostic test, microRNA, normal distant mucosa, brushing

## Abstract

MicroRNAs have recently been proposed as non-invasive biomarkers in Oral Squamous Cell Carcinoma (OSCC). The aim of this study was to analyze the expression of a panel of miRNAs in epithelial cells collected by oral brushing from OSCCs from regenerative areas after OSCC surgical resection and from their respective normal distant mucosa. Oral brushing specimens were collected from 24 healthy donors, 14 OSCC patients with specimens from tumour and normal distant mucosa, and from 13 patients who had OSCC resection, with samples from regenerative areas after OSCC resection and normal distant mucosa. Expression levels of eight targets (miR-21, miR-375, miR-345, miR-181b, miR-146a, miR-649, miR-518b, and miR-191) were evaluated by real-time Polymerase Chain Reaction (PCR). A highly significant between-group difference was found for miR-21 (F = 6.58, *p* < 0.001), miR-146a (F = 6.974, *p* < 0.001), and miR-191 (F = 17.07, *p* < 0.001). The major difference was observed between samples from healthy donors and from OSCC brushing, whereas no significant differences were observed between areas infiltrated by OSCC and their respective normal distant mucosa. Furthermore, altered expression of miR-146a and miR-191 was also observed in regenerative areas after OSCC resection. Conclusions: Oral brushing could be proposed as a noninvasive method to study microRNA expression in oral mucosa in OSCC patients.

## 1. Introduction

The incidence of second neoplastic manifestations in patients surgically treated for Oral Squamous Cell Carcinoma (OSCC) is high, ranging from 17% to 30% [1], and comprises both synchronous and metachronous tumours that develop before and at 6-month intervals between primary tumours, respectively [2]. Second manifestations may be distant or arise in the same area as the primary tumour.

Clinical monitoring and periodical follow-up in OSCC surgical patients are of primary importance for the early detection of recurrences or second primary cancers. Diagnosis is relatively simple when the lesion is clearly evident, but diagnosis is challenging in the early phase of neoplastic transformation. An oral biopsy could improve the detection of OSCC precursor lesions. Nevertheless, despite being a minor surgical procedure, sometimes patients will decline an oral biopsy due to postoperative pain. For this reason, a noninvasive screening tool would be easily accepted by patients and very helpful to detect the early development of tumour recurrence.

For almost four decades, changes in tumour suppressor protein-coding and/or oncogenes have been thought to be the main drivers of tumour development [3,4]. However, the discovery of non-coding RNAs suggested a more complex scenario [5]. MiRNAs, 18–25 nucleotide-long non-coding RNA molecules, have revolutionized our understanding of the modulation of gene expression. More than 1000 miRNAs have been identified in humans (miR-base; Available online: http://microrna.sanger.ac.uk) [6]. Highly ubiquitous and largely conserved across species, miRNAs regulate gene expression post transcriptionally by base pairing, usually imperfectly, to the 3′-untranslated region of a related mRNA [7]. A single miRNA can regulate the expression and/or function of hundreds of target mRNAs and proteins and control several biological processes (e.g., cell proliferation, differentiation, migration, apoptosis, and signal transduction) [8,9,10].

Overexpression of certain (as miR-17-92) miRNAs in carcinogenesis could result in downregulation of the tumour suppressor gene, while underexpression of certain (as let-7) miRNAs could cause oncogene upregulation [11]. This indicates that miRNAs may act as either tumour suppressors or oncogenes. Furthermore, different miRNAs have been identified in cancer related genomic regions, such as fragile sites and sites with frequent loss of heterozigosity [12]. A deregulation of several miRNAs (as miR-21, miR-31, let-7, or miR-155) is correlated with various tumour characteristics and prognosis in some cancers, including OSCC [7,13]. Specifically, several authors showed a relationship between an altered expression of miR-21, miR-345, miR-181b, miR-146a, and miR-375 and oral cancer tumorigenesis and progression [14,15,16,17,18,19,20]. Cervigne et al. revealed the presence of a dysregulation of the aforementioned miRNAs, together with miR-649 and miR-518, in Oral Potentially Malignant lesions (OPML) and in OSCC [14]. Other authors identified a relationship between an altered expression of miR-21, miR-146a, miR-181b, and miR-375 and OSCC aggressiveness and poor prognosis [16,18,19,21,22]. The majority of these studies identified an altered miRNA expression in neoplastic and preneoplastic lesions starting Formalin Fixed Paraffin Embedded (FFPE). A miRNA dysregulation has also been detected in the field of cancerization [23,24,25,26]. MiRNA-based molecular analysis could improve histopathological analysis through early detection of neoplastic transformation by monitoring the field effect in oral cancerization, a factor carrying major implications in OSCC prognosis.

MiRNAs possess unique properties which make them promising diagnostic and prognostic markers to be applied as oral cancer screening tests starting from noninvasive specimen collection avoiding postoperative pain. Firstly, miRNAs are abundantly expressed in OSCC and in control tissues. Their isolation and quantification seem to be easy, convenient, and reproducible [27,28] and several OSCC-related miRNAs may be isolated from body fluids or exfoliated cells [5].

Aberrant miRNA expression in OSCC has been identified in saliva samples [29,30,31,32,33,34,35,36,37,38,39,40,41,42], but these data do not discriminate between circulating and local production of miRNA. A different noninvasive method to obtain miRNA is brush cytology, which can collect exfoliating cells of the oral cavity. Unlike saliva sampling, brush cytology can collect cells from a specific surface of the oral cavity and from different sites of the oral cavity in the same patient [43,44,45].

The present study aimed to determine if epithelial cells collected by oral brushing could be a suitable biological source to explore miRNA expression levels of specific areas of oral mucosa. We evaluated whether alteration of cancer-related miRNAs can be detected in OSCC exfoliated cells, in the distant clinically normal mucosa of OSCC patients, and in the regenerative area after tumour resection.

## 2. Results

**Figure 1:**
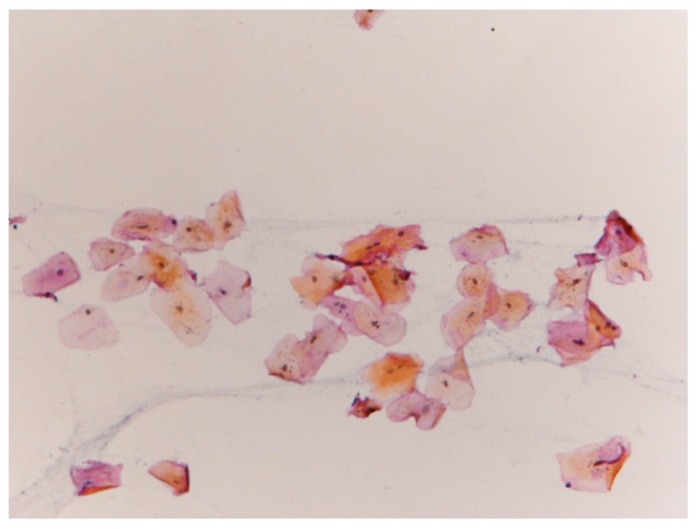
PAP smear evaluation showed the presence of keratinocytes of the upper and medium layer within the brush.

A total of 78 oral brushing specimens from 51 patients were collected for the study. Specimens were classified in five different groups as detailed in the methods section. Cytological examination of collected cells revealed only the presence of keratinocytes of the upper and medium layer (Figure 1).

Expression levels of a panel of eight miRNAs were analysed by real-time PCR. All specimens provided a suitable amount of total RNA, ranging from a minimum of 550 up to 6700 ng (median = 1800, interquartile range 1100–3000). The assay for miR-181b and miR-375 did not detect the target in 31/78 (40%) and 48/78 (62%) samples, respectively, and thus data obtained from these assays were not considered for further statistical analysis. This could be related to either low assay efficiency or weak expression levels of these miRNAs.

In a preliminary step, we investigated whether smoking can influence miRNA expression levels in exfoliated oral mucosa cells. No evidence of differential expression was found between smokers and nonsmokers, either considering the whole sample (Table 1), or within each group of patients.

**Figure 2:**
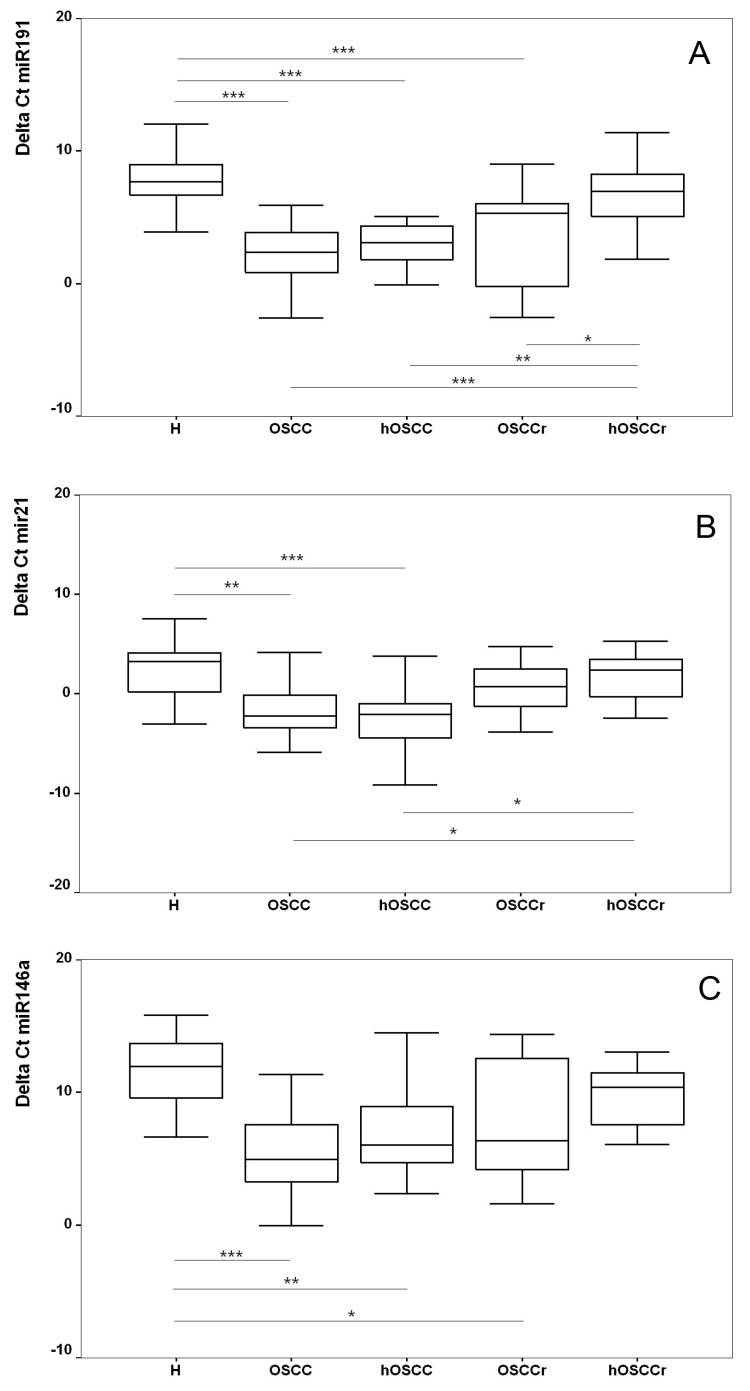
Expression levels of miRNAs. The box plots report the normalized expression levels of miR-191 (**A**), miR-21 (**B**), and miR-146a (**C**). Only miRNAs showing different expression among patient groups are included. Normalized expression levels are expressed as **∆^−^*^C^*^t^**; lower **∆^−^*^C^*^t^** values signify higher expression levels. The box represents the interquartile range of the **∆^−^*^C^*^t^**. The line across the box indicates the median, while the whiskers extend to the highest and lowest values. Level of significance in post hoc analysis for differences between groups were reported (* if *p* value < 0.05; ** if *p* value < 0.01; *** if *p* value < 0.001). H, healthy donor; OSCC, Oral Squamous Cell Carcinoma; hOSCC, distant healthy mucosa of OSCC patients; OSCCr, OSCC in remission; hOSCCr, distant healthy mucosa of OSCC patients in remission.

Next, we tested whether each miRNA was differently expressed in the defined specimen groups, i.e., OSCC, distant healthy mucosa of OSCC patients (hOSCC), OSCC in remission (OSCCr), distant healthy mucosa of OSCC patients in remission (hOSCCr), and healthy donors (H). The one-way Analysis of Variance indicated differential levels of expression among groups for three miRNAs: miR-21 (F = 6.58, *p* < 0.001), miR-146a (F = 6.97, *p* < 0.001), and miR-191 (F = 17.71, *p* < 0.001), whereas no differences were found concerning miR-345 (F = 2.14, *p* = 0.085), miR-518b (F = 0.44, *p* = 0.770), and miR-649 (F = 1.84, *p* = 0.142). A post hoc analysis was performed to compare expression levels of differentially expressed miRNAs between groups. It was found that miR-21, miR-191, and miR-146a were overexpressed in OSCC with respect to healthy donors (Figure 2). Surprisingly, expression levels in distant healthy mucosa of OSCC patients (hOSCC group) was elevated as well and not different with respect to tumours. Elevated expression of miR-146a and miR-191 was also observed in regenerative areas after tumour removal (OSCCr group) but not in distant healthy mucosa (hOSCCr group) (Figure 2, panel A and C).

The calculated expression fold changes in OSCC with respect to healthy donors were 50 (95% CI 12–211) for miR-191, 16 (95% CI 2–104) for miR-21, and 52 (95% CI 6–452) for miR-146a (Table 2).

## 3. Discussion

Oral mucosa brushing was recently proposed as an innovative noninvasive approach for OSCC diagnostic testing, potentially useful for early detection and assessing prognosis through the identification of sensitive and specific markers [46,47,48].

The present study used cytological oral brushing as a noninvasive sampling method to analyse a set of eight miRNAs (miR-21, miR-345, miR-518b, miR-649, miR-146a, miR-181b, miR-375, and miR-191) previously proposed for the early detection of Oral Potentially Malignant Lesions and OSCC [5,14]. The analysis recognized that miR-191, miR-21, and miR-146a were overexpressed in cells exfoliated from tumour lesions with respect to cells from healthy donors. The marked increase observed—with fold changes ranging from 16 (95% CI 2–104) for miR-21 to 52 (95% CI 6–452) for miR-146a—suggests a potential role for their use as diagnostic biomarkers.

Furthermore, in the study, we compared miRNA expression between samples collected from OSCC and regenerative areas after OSCC resection as well as clinically normal mucosa in areas distant from both tumour lesions and previously treated tumours. Interestingly, we found that all the miRNA overexpressed in OSCC showed similar expression levels even in normal distant mucosa from OSCC patients, suggesting the presence of a genetically altered field.

Different mechanisms could be responsible for an altered miRNA expression profile in clinically normal mucosa distant from OSCC. Oral brushing is a sampling method aimed at collecting exfoliated epithelial cells from a selected area prone to collect contaminating material, such as migrating cells and extracellular vesicles contained in saliva. However, the sampling protocol adopted in this study was designed to minimize any unwanted carry-over contaminants.

The altered miRNA expression profile detected in clinically normal mucosa distant from OSCC could also be explained by Slaughter’s model of “field cancerization” [49]. According to this model, a number of cells in areas without any sign of a tumour could have been genetically altered by a carcinogenic agent [47,50,51,52,53,54]. However, assuming that the mucosa is predisposed to carcinogenesis due to previous exposure to exogenous genotoxins, an important clinical implication is that fields should also remain altered after primary tumour resection. This may lead to new cancers that clinicians currently refer to as “a second field tumour” [1,54]. However, we observed that miRNA expression in distant mucosa subsides after OSCC resection to levels that did not significantly differ from those of healthy donors. Based on this evidence, we could hypothesize a sort of transient field cancerization effect mediated by the OSCC. In this model, the primary tumour can alter the genetic status of the oral mucosa possibly through a still-undetected diffusible element. Dysregulation of the oral mucosa could represent a preactivated status more sensitive to additional hits that could lead to transformation events.

An altered miRNA expression pattern in oral brushing specimens from normal-appearing buccal mucosa was observed in patients harbouring lung cancer [25]. The authors postulated a possible field cancerization effect due to higher tobacco smoking in patients. The level of miRNA modulation (fold change) was modest; in this case 3-fold lower. We observed a much higher modulation level of miRNAs in OSCC patients, up to 52-fold, whereas no significant association was found between miRNA expression and smoking in line with previous reports [23,24,25,26].

Irrespective of possible explanations, the fact that OSCC patients showed higher expression levels of miR-191, miR-21, and miR-146a in brushing from both tumour and normal mucosa implies that the analytic comparison of specimens from the same patients may have a limited relevance for OSCC diagnosis.

The present study also observed that both miR-191 and miR-146a showed altered expression in the regenerative area of patients surgically treated for OSCC. This finding could support the hypothesis of a field cancerization effect detectable after surgical resection in close proximity to the index tumour. On the other hand, we cannot exclude that these alterations are related to the regenerative process itself, such as active cell proliferation or inflammation. In this regard, miR-21 expression levels did not differ from those of controls and hence appear more specifically related to an effectively transformed status.

MiR-21 is one of the most extensively studied miRNAs in oral and head and neck squamous cell carcinomas and many other tumours. Experimental evidence demonstrated that miR-21 is able to promote cancer through inhibition of several genes, such as phosphatase and tensin homologue (PTEN) on chromosome 10, tropomyosin-1 (TPM1), and programmed cell death-4 (PDCD4) [55,56]. Previous studies in OSCC have shown that overexpression of miR-21 is strongly correlated with the progression of OPML to invasive OSCC [14]. In addition, miR-21 overexpression seems to be a poor prognostic marker, being associated with shorter survival [21,55].

MiR-191 has been found dysregulated in a large number of different human tumours, including colorectal cancer, breast prostate cancer, and acute myeloid leukemia [57,58]. A recent paper by Gombos et al. identified an altered miR-191 expression in OSCC samples with respect to normal tissues [59]. Target genes of the mature miRNA sequence have not been fully characterized, but colorectal cancer appears to promote tumorigenesis through targeting of CCAAT/enhancer binding protein beta and tumour invasion by directly targeting tissue inhibitors of metalloprotease 3 (TIMP3) [57,60].

MiR-146a deregulation has been found in oral cancer, though reports on OSCC are contradictory. MiR-146a has been demonstrated to be overexpressed in oral cancer and to enhance tumorigenesis in both in vitro and in vivo mouse xenograft models [17,61]. The same group suggested that a G to C polymorphism (rs2910164) in pre-miR-146a was prevalent in OSCC patients with lymph node metastasis [61]. However, findings reported by Palmieri et al. and a recent metanalysis indicated that the rs2910164 polymorphism is not associated with OSCC progression [62,63].

The oncogenic functions of miR-146a were associated with concomitant downregulation of the IL-1 receptor associated with kinase 1 (IRAK1), TNF receptor-associated factor 6 (TRAF6), and NUMB endocytic adaptor protein (NUMB) [61]. Shi et al. recently analysed OSCC cell lines and identified the Sox2 mRNA as a potential new target for miR-146a [18]. Sox2, a member of the SOX (SRY-related high mobility group box) family, was initially described in the context of embryonic stem cell pluripotency [64]. Recently, Du et al. showed a close relationship between poor prognosis in oral tongue squamous cell carcinoma and Sox2 expression [65]. Silencing Sox2 induced mesenchymal–epithelial transition [66], which involved the localization of miR-146a within the layers of normal stratified squamous epithelium in oral mucosa [61,67].

Here, we demonstrated that oral brushing is a simple and noninvasive method able to collect specimens useful for miRNA quantification by PCR. This approach confirmed that miRNAs are dysregulated in OSCC but also that the OSCC is able to dysregulate miRNAs in normal mouth mucosa. In turn, we reported data supporting that miRNA-based noninvasive diagnostics is technically feasible and data supporting the field cancerization theory. On the other hand, identification of a set of miRNAs specifically expressed in OSCC would facilitate diagnostic testing. The identification of optimal biomarkers specific for each different clinical need—early diagnosis, tailored cure, specific prognosis—could be the subject matter of our next investigations.

## 4. Material and Methods

### 4.1. Ethics Statement

All clinical investigations were conducted according to the principles expressed in the Declaration of Helsinki. The study was approved by the local Ethics Committee (study number 14092, protocol number 899/CE, 23 February 2015). All information regarding the human material used in this study was managed using anonymous numerical codes.

### 4.2. Sample Collection

The study included patients referred to the Department of Oral Sciences and Section of Maxillo-facial Surgery at S. Orsola-Malpighi University Hospital, Bologna, from March 2015 to July 2016.

Oral brushing specimens were collected from three groups of patients. The first group comprised 14 consecutive patients with a diagnosis of OSCC at the time of collection. In this group, two different brushing specimens were collected: the first within clinically evident OSCC and the second in clinically normal distant mucosa (opposite cheek). Oral brushing specimens were always picked before incisional biopsy and samples were admitted in the study population only after diagnostic confirmation of OSCC by histological examination.

Histological examination was performed on a blinded basis at the Department of Biomedical and Neuromotor Sciences, M. Malpighi Section of Anatomic Pathology at Bellaria Hospital, University of Bologna, Italy. Two pathologists (M.P.F. and S.A.) examined all samples and for discordant cases, a common diagnosis was obtained upon discussion at multi-head microscope. Histological diagnoses were performed following World Health Organization (WHO) criteria [68].

The second group was composed of 13 consecutive patients who had completed OSCC treatment at least 6 months prior to brushing collection and have had no recurrence of cancer since then. This group included patients with OSCC in remission (OSCCr) who were enrolled during routine follow-up visits after primary oral cancer surgical treatment.

All 13 enrolled patients underwent primary tumor resection and neck dissection according to international guidelines [69]. Reconstruction using local or microvascular free flaps was performed in relation to the stage of disease. Post-operative radiation therapy was considered an exclusion criterion in the present study. Two different samples were also collected from this group: the first within the regenerative area after OSCC resection (with or without presence of a skin graft used for tissue reconstruction after OSCC resection) and the second in the clinically normal distant mucosa (opposite cheek).

The third group included 24 healthy donors. In this group, only one oral brushing sample was collected from similar areas to the OSCC group. The total study population comprised 78 oral brushing samples:

**Group OSCC**: 14 specimens collected from OSCC lesions

**Group hOSCC**: 14 specimens collected from healthy distant mucosa in OSCC patients

**Group OSCCr**: 13 specimens collected from regenerative area after OSCC resection

**Group hOSCCr**: 13 specimens collected from healthy distant mucosa in OSCC surgically treated patients

**Group H**: 24 specimens collected from healthy donors

Table 3 provides information on patient age, sex distribution, smoking habits, and site of brushing specimen of each group of patients.

### 4.3. Oral Brushing Method

A cytobrush (GPS Medical, Mozzo (BG), Italy) was used to collect exfoliated cells from oral mucosa as previously described [47,48]. In OSCC lesions and in regenerative areas after OSCC surgical resection, all lesion surfaces were gently brushed repeatedly five times. Cell collection was always performed before incisional biopsy and before any local anaesthetic injection. After brushing, each cytobrush specimen was placed in a 2 mL tube containing RNAlater™ (Sigma Aldrich, Inc., St. Louis, MO, USA) and stored at −20 °C for cell and miRNA preservation.

A cytological smear test was performed on 10 randomly selected specimens for a qualitative cell evaluation of the sampling collection.

### 4.4. RNA Isolation and cDNA Synthesis

Total RNA, including miRNAs, was purified by the Tri Reagent^®^ technique (Ambion Inc., Austin, TX, USA). All procedures were performed according to the manufacturer’s recommendations. Tubes containing the cytobrush were supplemented with 500 μL of chilled PBS and centrifuged (5000× *g*, 10′). Then, the brushes were discarded and the samples were centrifuged again (6000× *g*, 30′). The supernatant was discarded and 1 mL Tri Reagent^®^ Solution (Ambion Inc., Austin, TX, USA) was added to the cell pellet and incubated at room temperature for 10′, then 200 μL of chloroform was added and homogenized to each sample. After centrifugation for 15′ at 12,000× *g* (4 °C), the upper aqueous layer containing the total RNA was transferred to a new sterilized tube, 500 μL of 100% isopropanol was added, then the tube was homogenized by inversion and incubated at room temperature for 10′ and centrifuged at 12,000× *g* for 10′ at 4 °C.

The RNA pellet was washed with 1 mL of 75% ethanol. The mixture was vortexed briefly then centrifuged at 12,000× *g* for 5′ at 4 °C. The RNA pellet was air-dried and resuspended in 11 μL of RNase-free water.

The purity and concentration of recovered RNA were determined with the NanoDrop 2000 spectrophotometer (Thermo Fisher Scientific, Wilmington, DE, USA). Up to 800 ng of total RNA including miRNAs was reverse-transcribed using the NCode™ VILO miRNA cDNA Synthesis Kit (Invitrogen, Carlsbad, CA, USA) according to the manufacturer’s protocol in a volume of 25 μL. A 1:4 dilution of this reaction was used as the template for real time PCR.

### 4.5. miRNA Expression

Expression levels of miRNAs were evaluated by real-time PCR. Eight targets were evaluated, i.e., miR-191, miR-21, miR-375, miR-345, miR-181b, miR-146a, miR-649, and miR-518b, while the small-nucleolar RNA RNU44 was used as an endogenous reference for data normalization.

PCR analyses were performed using the SYBR^®^ Green JumpStart™ TaqReadyMix™ (Sigma Aldrich, Inc.) with miRNA-specific forward primers modified with locked nucleic acid (LNA) substitutions for increasing specificity and discriminating between miRNAs with single-base and different nucleotide sequences [46] and a universal reverse qPCR primer (provided in the NCode VILO miRNA cDNA synthesis kit) according to the manufacturer’s protocol using the ABI PRISM 7500 machine (Applied Biosystems, Waltham, MA, USA). Briefly, the reaction was performed in a total volume of 20 μL, including 3 μL of template, for 38 thermal cycles and subsequent melting analysis of the amplimers. Amplification efficiencies of the reference and targets were close to 100%. All targets of each sample, as well as controls, were processed in the same reaction plate in triplicate. The mean Ct of each target miRNA was compared to the reference RNU44; the obtained **∆^−^*^C^*^t^** were used as quantitative data for statistical comparison of sample groups. The fold change of target gene expression in a patient group relative to the healthy donors was calculated with the 2^−ΔΔ*C*t^ formula.

### 4.6. Statistical Analysis

Data processing and statistical analysis were performed using SPSS software. One-way ANOVA was used to compare the miRNA expression level between groups of specimens (OSCC, hOSCC, OSCCr, hOSCCr, and H, as described above) and to determine whether any of those means were significantly different from each other. Data for the analysis were the ∆^−*C*t^, i.e., logarithms of relative expression values. When results were statistically significant (*p* value < 0.05), Dunnet’s post hoc test was performed to explore which specific groups of patients differed from each other.

## 5. Conclusions

This pilot study indicates that miRNAs can be eligible as target biomarkers for noninvasive OSCC diagnostic testing. Indeed, we observed that some OSCC-related miRNAs, namely miR-21, miR-191, and miR-146a, were significantly overexpressed in OSCC cells facing the oral cavity. Although the data must be considered with caution due to the relatively small size of the cohort, our study disclosed an altered miRNA profile expression also in areas distant from OSCC. This has important implications in the mechanistic explanation for the high OSCC recurrence risk. The identification of optimal target miRNAs for early diagnosis, tailored cure, and specific prognosis deserves additional research efforts.

## Figures and Tables

**Table 1:** Comparison of expression levels of selected miRNAs between smokers and nonsmokers.

miRNAs	Smoke	Mean ∆^−^*^C^*^t^	SD	Student *t*	*p* Value
miR-191	yes	4.9	3.5	0.5	0.6
	no	4.4	3.7		
miR-21	yes	−0.3	3.7	−0.7	0.5
	no	0.4	4.2		
miR-345	yes	3.2	3.5	−0.5	0.6
	no	3.7	3.7		
miR-146a	yes	8.6	4.6	0.2	0.9
	no	8.1	4.1		
miR-649	yes	8.7	1.7	−0.3	0.7
	no	9.0	2.9		
miR-518b	yes	5.5	5.2	−1.2	0.2
	no	7.0	4.2		

**Table 2:** Dunnet post hoc test for differently expressed miRNAs. Expression levels of miRNAs in each group of specimens were compared with healthy controls. Entries in boldface and with asterisk (*) indicate statistically significant *p* values.

miRNAs	Reference	Test Group	*p* Value	∆∆^−^*^C^*^t^	Fold Change
mir-191	H	OSCC	< 0.001 *	−5.7 (95% CI −7.7; −3.6)	50 (95% CI 12; 211)
	H	hOSCC	< 0.001 *	−4.7 (95% CI −6.8; −2.7)	27 (95% CI 6; 111)
	H	OSCCr	< 0.001 *	−4.2 (95% CI −6.4; −2.1)	19 (95% CI 4; 82)
	H	hOSCCr	0.632	−1.0 (95% CI −3.2; 1.2)	2 (95% CI 0.4; 9)
mir-21	H	OSCC	0.002 *	−4.0 (95% CI −6.7; −1.3)	16 (95% CI 2; 104)
	H	hOSCC	< 0.001 *	−4.5 (95% CI −7.3; −1.7)	23 (95% CI 3; 161)
	H	OSCCr	0.770	−1.1 (95% CI −3.9; 1.7)	2 (95% CI 0.3; 15)
	H	hOSCCr	0.992	−0.4 (95% CI −3.3; 2.5)	1.3 (95% CI 0.2; 10)
mir-146a	H	OSCC	< 0.001 *	−5.7 (95% CI −8.8; −2.6)	52 (95% CI 6; 452)
	H	hOSCC	0.003 *	−4.4 (95% CI −7.6; −1.2)	22 (95% CI 2; 198)
	H	OSCCr	0.020 *	−3.9 (95% CI −7.3; −0.5)	15 (95% CI 1.4; 154)
	H	hOSCCr	0.687	−1.6 (95% CI −53; 2.1)	3 (95% CI 0.2; 39)

**Table 3:** Clinical profile of population study.

Participants	N.	Sex	Mean Age	Smoker	Alcohol Consumption	T Stage	N Stage	Site of Brushing Specimen	
								First	Second
OSCC	14	5 M, 9 F	68.15 ± 13.09	5 Yes 9 No	2 Yes 12 No	6 T1 6 T2 2 T4	12 N0 2 N2	5 Cheek 5 Gingiva 4 Tongue	Opposite cheek
OSCCr	13	4 M, 9 F	58.9 ± 9.38	1 Yes 12 No	1 Yes 11 No	3 T1 6 T1 1 T3 3 T4	9 N0 1 N1 2 N2 1 N3	5 Tongue 3 Gingiva 4 Cheek 1 Floor of mouth	Opposite cheek
Healthy donors	24	12 M, 12 F	50.4 ± 18.6	8 Yes 16 No	3 Yes 21 No			8 Cheek 8 Gingiva 8 Tongue	
